# Structural Insights into the Human Astrovirus Capsid

**DOI:** 10.3390/v13050821

**Published:** 2021-05-01

**Authors:** Matthew Ykema, Yizhi J. Tao

**Affiliations:** Department of BioSciences, Rice University, Houston, TX 77005, USA; mry3@rice.edu

**Keywords:** astrovirus, capsid, structure, crystallography, virus maturation

## Abstract

Astroviruses (AstVs) are non-enveloped, positive single-stranded RNA viruses that cause a wide range of inflammatory diseases in mammalian and avian hosts. The T = 3 viral capsid is unique in its ability to infect host cells in a process driven by host proteases. Intercellular protease cleavages allow for viral egress from a cell, while extracellular cleavages allow for the virus to enter a new host cell to initiate infection. High-resolution models of the capsid core indicate a large, exposed region enriched with protease cleavage sites. The virus spike protein allows for binding to target cells and is the major target for naturally occurring and engineered neutralizing antibodies. During maturation, the capsid goes through significant structural changes including the loss of many surface spikes. The capsid interacts with host membranes during the virus life cycle at multiple stages such as assembly, host cell entry and exit. This review will cover recent findings and insights related to the structure of the capsid and its function. Further understanding of the viral capsid structure and maturation process can contribute to new vaccines, gastric therapeutics, and viral engineering applications.

## 1. Introduction

Astroviruses (AstVs) are positive, single-stranded RNA (+ssRNA), non-enveloped viruses. The family *Astroviridae* consists of two genera, *Mamastrovirus* and *Avastrovirus*, that infect a broad range of mammalian and avian hosts, respectively [1,2]. Classical human astroviruses (HAstVs) are classified into eight serotypes (i.e., HAstV-1 to 8), which are known to cause mild to severe gastroenteritis, usually in small children [2,3]. However, there have been recent cases of emerging HAstVs from two new clades, VA and MLB, that are associated with more severe gastroenteritis and neural pathogenesis [4,5,6]. This is especially interesting because animal AstVs are known to cause various inflammatory diseases in their hosts, ranging from gout in chickens, encephalitis in cows, and a degenerative neural infection in minks [7,8,9]. Furthermore, advances in environmental sampling and DNA sequencing technology indicate an increasingly expansive range of hosts. An earlier review by Arias and DuBois has gone into detail about the AstV capsid structure and its roles in virus infection and pathogenesis [10]. The goal of this review is to highlight the most recent findings about the structure and function of the AstV structural proteins and discuss future directions that are urgently needed to address outstanding research questions.

## 2. Astrovirus Capsid Maturation Is a Host-Driven Process

**Figure 1:**
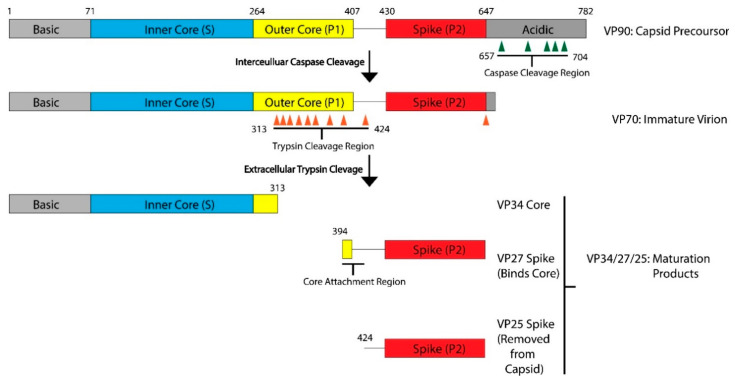
Maturation Process Modeled with HAstV-8. After translation, the viral capsid protein forms the VP90 precursor. The acidic domain is cleaved by caspases along sites in the acidic domain, resulting in the VP70 immature virion. This VP70 virion exits the cell, where it is exposed to extracellular proteases. There are multiple trypsin cleavage sites in the P1 outer core domain, leading to the generation of the VP34 core, VP27 spike, and VP25 spike domains. These three products, and possibly some of the digested peptides from the outer core domain, assemble into the VP34/27/25 mature capsid. VP27 can form a homodimer and associate with the VP34 core domain. VP25, which has the core attachment region cleaved, is not present in the mature particle. Domains and regions are numbered according to the sequence of HAstV-8, but the domain architecture and many of the protease cleavage sites are conserved across HAstV-1 to 8 serotypes. Adapted from [16,17].

The AstV capsid is first assembled from a polyprotein expressed from the open reading frame 2 (ORF2) located at the 3′-end of the viral RNA genome. ORF2 is translated from a subgenomic RNA, and the lack of sequence similarity in ORF2 to other enteric viruses including picornaviruses and caliciviruses led to the classification of AstVs into a separate family [10]. AstV ORF2 is translated into a five-domain protein called VP90, which is approximately 90kD in size. From N-terminus to C-terminus, the VP90 polypeptide folds into the basic domain (~80 aa), the inner core (S, ~180 aa), the outer core (P1, ~150 aa), the spike (P2, ~200 aa) and the acidic domain (~140 aa) (Figure 1). After translation, 180 subunits are assembled into the T = 3 icosahedral capsid and form the VP90 state of the virus. In cellular studies, VP90 mostly associates with the membrane fractions [11]. VP90 can dissociate from the membranes and become soluble after cleavage by proteases, specifically caspases from virus-induced apoptosis [11,12,13]. There are several candidate caspase cleavage sites in the C-terminal acidic domain, and this maturation occurs over a period of 10–20 min after translation [14]. The involvement of host caspases in viral protein cleavage and processing is a process that is not unique to AstVs, having been reported in other viruses including parvovirus, human papillomavirus and SARS-coronavirus [15]. Unlike these other viruses, other than the removal of the ~20 kD acidic domain and dissociation from membranes, very little is known about the role or importance of these caspase cleavages in the transition of the VP90 state to the VP70 state. It is possible that VP90 may play a role in viral capsid assembly or help suppressing host immune response by sequestering the viral capsid and RNA to host membranes, but further studies are needed to test these hypotheses.

After the removal of the acidic domain by caspases, the astrovirus capsid is converted into the VP70 state. The virus can now dissociate from cellular membranes and exit the cell [18]. AstV infection increases cell-to-cell and tight junction permeability without inducing cell death, indicating that the VP70 exits from intestinal cells in a non-lytic fashion [19]. However, the capsid properties and host membrane remodeling process that allow for non-lytic exit are unknown. Very few functional studies have been conducted on the VP70 state of the virus, but it appears to be a transitory state that is stable in the extracellular environment. Astrovirus with the VP70 capsid is non-infectious, however, and infectivity requires a maturation step that involves another round of proteolytic processing by host extracellular proteases. In vitro, the VP70 capsid can be matured by trypsin to the VP34/27/25 state [20,21]. On SDS-PAGE, mature astrovirus capsid produces three bands at 34 kD, 27 kD and 25 kD that roughly correspond to the inner core domain, the longer and the shorter version of the spike domain, respectively. The VP34 core is separated from the VP27/25 spike domains [20], with a gap of approximately 80 amino acids in between that is unaccounted for in the SDS-PAGE [16]. Therefore, the VP27 spikes are retained in the mature astrovirus particle due to noncovalent inter-domain binding rather than covalent polypeptide linkage, with the VP25 dissociating from the mature viral capsid during the trypsin cleavage. Functionally, trypsin treatment increases the infectivity of the viral particles by up to 10^5^ fold. The molecular determinant of the trypsin-imparted infectivity is unknown, but proposed to be related to the loss of 60 capsid spikes and freeing of a possible functional motif(s) on the capsid [20,21]. 

In summary, the AstV capsid maturation process is quite dynamic, resulting in the virus moving from a non-infectious intracellular state (VP90) to a primed extracellular state (VP70), and finally to a fully mature and infectious particle (VP34/27/25). This process is driven by the host proteases, which may have a major role in virus–host interactions and tissue/cellular tropism. There are still many unanswered questions related to viral maturation process, such as the level of conservation across strains, capsid functions enabled by maturation, and the nature of protease factors driving maturation in different hosts or different tissue environments. 

## 3. Capsid Structures and Modelling Provide Insights into Serotype Variability

**Figure 2:**
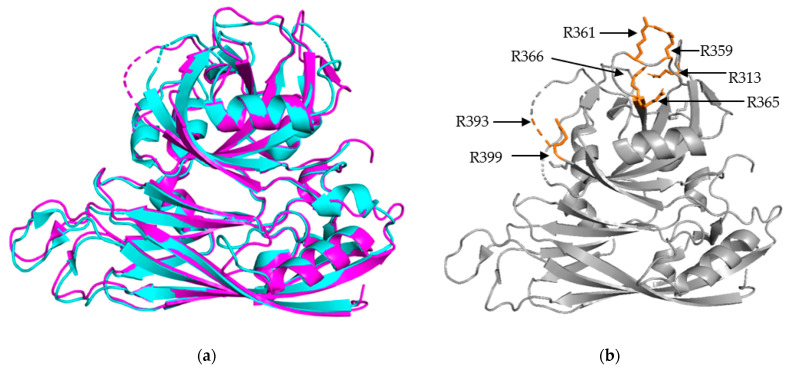
Structural Alignment of HAstV-1 and HAstV-8 core domains. (**a**) Structural alignment of HAstV-1 aa 80-429 (cyan) and HAstV-8 aa 71-415 (magenta) indicates a high level of structural similarity. (**b**) Trypsin cleavage sites, labeled in orange, for HAstV-8 (grey) are clustered on the exterior surface of both core domains. R393 is located in a structurally disordered loop, so the arrow is used to indicate its approximate position. Structures adapted from [16,23].

**Figure 3:**
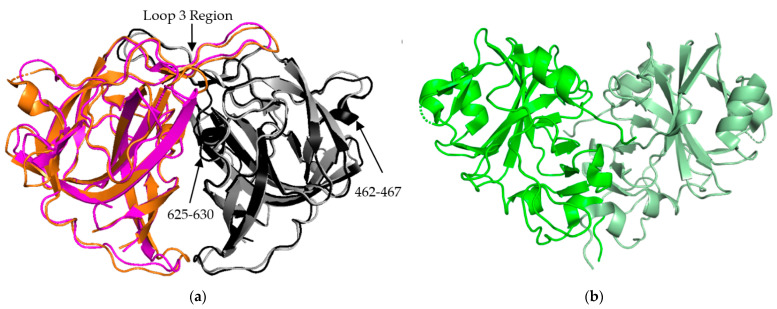
AstV Spike Structures. (**a**) Structural alignment of HAstV-2 aa 429-645 spike dimer (cyan/black) to HAstV-8 aa 415-646 spike dimer (magenta/grey) indicates a high level of sequence similarity outside of domains from 462-467 and 625-630. The loop 3 region maintains the dimeric state of the spike. (**b**) Structure of TAstV2 aa 421-724 spike dimer (dark green/light green) shows a significantly different, V-shaped architecture when compared to the human virus structures. Structures adapted from [23,24,25].

X-ray crystallography studies have produced several high-resolution structures that reveal an extensive amount of information about individual domains of the AstV capsid and the level of variations across strains (Figure 2 and Figure 3). These structures cover two main regions of the virus, the S/P1 core domains and the P2 spike domain. Currently, there are no high-resolution structures of the N-terminal basic or C-terminal acidic domains. The N-terminal basic domain is rich in lysine and arginine residues but is predicted to have very little secondary structure. The C-terminal acidic domain is predicted to have a number of short alpha helixes, but the rest of the domain is unstructured and has regions high in aspartate and glutamate [22]. It has been difficult to obtain crystal structures of the N- and C-terminal domains due to their high charge content and the lack of secondary structures. 

### 3.1. S/P1 Core Domain

**Figure 4:**
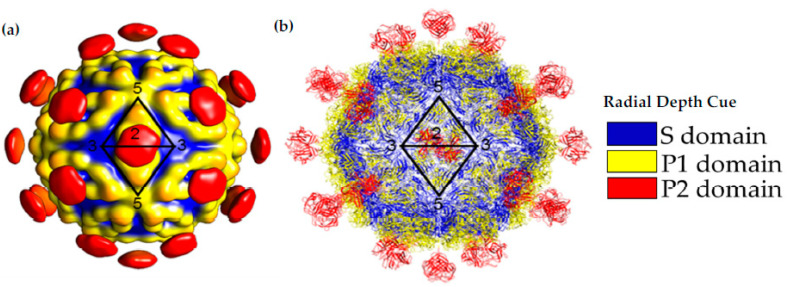
Capsid Structure of the mature HAstV-8 VP34/27/25 Particle. (**a**) Cryo-electron microscopy map at of resolution of ~25 Å of the mature viral capsid colored by radial depth cue from blue to yellow to red. Dimeric spikes are seen on the 2-fold axis, revealing the 3- and 5-fold axes of the core. (**b**) HAstV-8 crystal structures for the spike and core modeled into a capsid. The S, P1 and P2 domains are colored in blue, yellow and red, respectively. Adapted from [16,24,27].

The S/P1 core domain structures have been solved for two human astrovirus serotypes, HAstV-8 and HAstV-1 [16,23]. These structures were solved to similar resolutions, 2.15 Å for HAstV-8 and 2.60 Å for HAstV-1. The two structures, aligned in Figure 2a, show that the S domain is mostly comprised of β sheets, forming a β barrel jelly roll fold, which is a common structural motif found in viral capsids [26]. The S domain forms the innermost layer of the protein capsid shell. The P1 outer core domain, as the name implies, is oriented away from the capsid core, and sits near the exterior of the virus. It forms trimeric “turret-like” protrusions on the outside, allowing for the arrangement of the P2 spike domain on top (Figure 4). The P1 domain is comprised of short β strands (e.g., six for HAstV-1 and seven for HAstV-8) and α helices (e.g., 3 for both HAstV-1 and HAstV-8), with structured/unstructured loops spaced in between. The high percentage of structured loops gives the P1 domain an interwoven layout. When the maturation process is taken into consideration, especially the trypsin-driven transition from VP70 to VP34/27/25, an interesting pattern emerges. In both structures, the trypsin cleavage sites are presented on the edges of the domain, making them accessible to the host proteases (Figure 2b) [16,23]. This is a prime example of how the structure can rationalize findings from functional and cell culture studies. 

### 3.2. P2 Spike Domain

The P2 spike domain structure has been solved for three human astroviruses, HAstV-1, HAstV-2, HAstV-8 (Figure 3a) [25,27,28]. Across these three astroviruses, the spike domain consistently forms a globular dimer, resulting in 90 spikes covering the VP70 capsid. When superimposed, HAstV-1 and HAstV-8 spikes show close resemblance, with the structure being mostly made up of a hydrophobic core with six β sheets (Figure 3a). HAstV-1 has a nearly identical structural similarity to HAstV-8 but was not shown in Figure 3a for the purpose of clarity. The spike interior interface is primarily driven by loop 3, which extends from the top of the spike from one subunit towards the other subunit. This creates a large surface resulting in a strong dimer interaction as indicated by stable dimer formation when the spike domain was expressed alone [23,24,25]. Most of the structural variability across these three human astroviruses appears to be on the exterior surfaces, such as the presence or absence of α helices in the amino acid 462–467 and 625–630 regions (Figure 3a) [28]. These small differences of sequence and secondary structure can significantly contribute to differences in antibody recognition.

The crystal structure of the turkey astrovirus 2 (TAstV-2) spike has also been determined (Figure 3b) [29]. The CPs of *Avastrovirus* subfamily are usually smaller than those of *Mammastrovirus* [29,30]. This reduction in size is found throughout the ORF2 sequence, but the majority of the variability occurs in the C-terminal 200–300 amino acids out of the ~700 amino acid sequence that are mapped to the spike domain [31,32]. TAstV2 spike also forms a dimer, but has a V-like shape, fewer β strands, and a number of alpha helixes along the inner dimer interface and the exposed regions, leading to a significantly different spike appearance (Figure 3b) [25]. This indicates that while astroviruses across both genera share the same functional role and similar overall structural folds, there could be significant structural and functional differences in the spike domain from strains that have not been studied. This could be especially relevant for the emerging MLB and VA strains of AstV, classified to the *Mammastrovirus* genotype 1 and 2, respectively, that appear to have different pathogenesis than the eight classical human astrovirus serotypes [33]. More investigation into the capsid proteins of avian astroviruses will allow for the establishment of molecular patterns across the two astrovirus genera. 

While there have been no new astrovirus structures solved using X-ray crystallography in the last four years, there have been several recent studies using existing structural information to model the evolution of AstV structures [34,35]. A large amount of sequencing data has been collected related to known AstV strains, which can be used to analyze amino acid variations in various regions in existing structures. This approach was taken with HAstV-1 sequences from around the world, which identified six key sites across the VP34/27/25 domains that suggest sub-lineages of the virus. Variability in these key sites also indicate that these regions are under strong selection by host and antibody responses [34]. It is anticipated that novel approaches in structure modeling can be used to predict key differences between classical and emerging strains of human astroviruses and can complement structural studies to better understand antibody responses and functions related to the viral capsid protein.

## 4. Capsid Architecture Changes during the Virus Life Cycle

When the human astrovirus capsid assembles, it is made up of 180 subunits and maintains its T = 3 architecture throughout its lifecycle. The VP90 state of the virus includes all five domains of the virus capsid protein (CP), with the C-terminal acidic domain somewhere at the exterior of the virus, allowing it be accessible to cellular membranes and caspases. The VP90 state of the virus has been difficult to isolate, but several studies have used recombinant systems to attempt to isolate this state of the capsid. A vaccinia virus system was used to express the HAstV-2 ORF2 sequence in BSC-40 and LLCMK2 cells, which showed the generation of aggregations of virus-like particles (VLPs) in the infected cells [36]. There has been another report where an insect cell/baculovirus platform was used to generate VLPs of HAstV-1 after making truncations to the N-terminal region of the VP90 [37]. At this point in time, no recombinant VLPs have been investigated using structural techniques, such as cryo-EM. These recombinant systems may provide an avenue for isolating theVP90 state for further study.

After maturation, VP70 state of the virus can egress from the cell. This has allowed for the isolation of the HAstV-8 VP70 particle from protease-inhibitor-treated cell culture lines and structural analysis by cryo-EM [27]. Due to the small dataset (i.e., less than 1000 particles) for data analysis, the structure is limited to ~25 Å resolution. This structure confirmed the T = 3 architecture of the capsid and showed 90 spikes on the capsid. This study was also able to isolate the HAstV-1 and HAstV-8 VP34/27/25 state of the virus by in vitro treatment of VP70 particles with trypsin or isolation of matured particles from cell culture, respectively. The structures of the mature particles revealed a massive change in the morphology of the capsid, with a loss of 60 of the 90 spike domains. The retained 30 spike domains were on the 2-fold axis of the capsid (Figure 4). Even though the model was of modest resolution, it was sufficient to show similarities to another T = 3 RNA virus, the hepatitis E virus, and allow for the fitting of crystal structures of individual domains (Figure 4) [16,23]. 

Post-maturation structural analysis indicated that the roughly 30 amino acid regions that present at the N-terminus of VP27 but are absent from VP25 are necessary to anchor the spike to the core domain, while VP25 on its own has no covalent or noncovalent interaction with the capsid core. This means that the spikes present in mature capsid are almost entirely VP27 [17]. It is unknown why the capsid spikes remain on the 2-folds but not on quasi-2-folds in mature capsid. It is possible that the 2-folds where the 30 remaining spikes are found can occlude the VP27 trypsin cleavage sites. It is also unknown if the cleaved VP25 has some role in the viral infection process, such as interacting with host cell components or acting as a decoy for the host immune response. Future high-resolution studies of both immature and mature astrovirus capsids can help visualize the subtle structural differences at pseudo-2-fold vs. icosahedral 2-fold symmetry axes that allow differential cleavage of the capsid spikes and how capsid structure changes upon trypsin-mediated maturation.

## 5. Capsid Receptor Binding and Entry

One of the outstanding questions regarding the AstV capsid is its ability to bind to host cell receptors to initiate viral entry. Along with protecting the viral genome, the AstV capsid must be able to bind with the host cell to initiate the entry process. Several earlier studies have looked into candidate receptors, with the spike domain having the properties to mark it as the candidate binding domain [38]. The structure HAstV-8 spike protein indicates the charged pairs of residues that act as a common motif to bind di/trisaccharide moieties, though this binding has not been verified [24]. After receptor binding, there have been a few cellular entry and uptake pathways that have been identified. Initial uptake appears to be driven by clathrin-mediated endocytosis, based upon RNA-interference studies in Caco-2 cells [39]. Very recent studies have indicated that protein disulfide isomerase A4 (PDIA4), an enzyme that assists in thiol-disulfide exchange and the unfolded protein response, is responsible for human astrovirus disassembly after entry [40,41]. An interesting observation is that different strains of astrovirus interacted with PDIA4 in different ways, with HAstV-1 and HAstV-8 spike proteins showing binding with PDIA4 and interference when PDIA4 was blocked, while HAstV2 had no PDIA4 association. This may indicate even the small sequence and structure variations in the HAstV-1 to 8 strains may lead to differences of receptor binding, entry, and uncoating, and such differences may be amplified in the more divergent VA and MLB strains.

## 6. Capsid/Membrane Interactions in Viral Assembly and Entry

As discussed above, membranes likely play an important role in AstV capsid assembly, but there is also evidence for capsid–membrane interactions that occur during viral entry and exit of an infected cell. In terms of viral entry, many non-enveloped viruses use a membrane-interacting domain or protein complex to allow for the delivery of the viral genome across membrane, such as other gastroenteric pathogens with the rotavirus VP4 being a prime example [42]. One of the first indicators that AstV may have unique interactions with cellular membrane was the observation that treatment of Caco-2 cells with active or inactive HAstV-1 particles resulted in a measurable increase in ionic flux indicative of gastroenteritis, without an increase in cell lysis or death [19]. However, the astrovirus capsid structure does not show any regions that are predicted to be transmembrane domains, amphipathic helices, or other elements that would disrupt cellular membranes that are found in other non-enveloped viruses known to disrupt cellular membranes during virus entry [16,24]. Virus-induced type I interferon response has been implicated in reducing the permeability effect, suggesting that the enhanced tight junction permeability in astrovirus-infected cells is a specific process driven by one or more of the viral proteins [43]. It is currently unknown what properties of the astrovirus capsid induce this membrane flux, as increases in flux are indicative of modifications to the epithelial barrier, but this activity may be driven by the mature VP34/27/25 capsid as part of its entry mechanism. 

AstV proteins may also play a role in the ability for the virus to exit the cell. While there is evidence the transition from the VP90 to VP70 state of the capsid allows for the virus to exit the cell in a non-lytic manner, other structural/functional relationships related to this transition are unknown [18]. A second factor that may drive viral exit has emerged from recent studies on the predicted 12 kD protein product of the ORFX region, which originates from a -1 frameshift at the beginning of the ORF2 sequence [44]. The ability for this region to be translated was found to be highly conserved across AstV variants based upon ribosome profiling. Functionally, the ORFX product has the ability to disrupt or permeabilize a number of host-cellular membranes, and may play a key role in viral assembly and egress from the cell, even though it may not be incorporated into AstV capsid [45]. Interestingly, even though the presence of ORFX and its function may be conserved, the properties of this product are extremely diverse across *Mammastrovirus* strains. The sequence ranges in length from 64 amino acids in murine AstVs to 148 amino acids in bat AstVs, with large variabilities in the chemical properties of the amino acids that make up the sequence. Some ORFX products are expected to have transmembrane regions in the C-terminus, while others have no predicted activity, indicating that membrane integration may not be necessary for the function. Structural studies on this protein product across the AstV family would be highly valuable to further understand the functions and underlying mechanisms of this novel ORFX product.

## 7. Capsid-Antibody Interactions

Serological analysis in pediatric populations across the United States indicate a prevalence of neutralizing antibodies ranging from 42% of the surveyed population to HAstV-3 to 94% to HAstV-1 [46,47]. This indicates a high burden and prevalence among pediatric populations, indicating a need for targeted antibody therapeutics in the absence of traditional vaccines. In a structural context, there have been a few interesting studies that have looked at how neutralizing antibodies relate to capsid domain, along with attempts to isolate and rationally engineer therapeutic antibodies [48]. So far, the only neutralizing epitope that has been structurally characterized is a quaternary structure dependent epitope on the side of the HAstV-2 spike [28]. This neutralizing monoclonal antibody is known as PL-2 (MAb PL-2). This antibody was first identified by its ability to bind the spike domain in HAstV-2 (VP26) and neutralize infection [49]. Using crystallography and mass spectrometry, the amino acid sequence of PL-2 Fab was deduced and a corresponding antibody single-chain variable fragment (scFv) was engineered and expressed with possible therapeutic potential. Cocrystal structure of HAstV-2 spike and PL-2 scFv further revealed the location of the epitope [28,48]. The mechanism of neutralization of PL-2 is to prevent receptor binding by the HAstV-2 spike. This information was then used to identify escape mutants to the PL-2 antibody, which could be traced to a single amino acid mutation at amino acid 463 in an outer loop of the capsid spike [50]. This body of work represents a template workflow for characterizing other monoclonal antibodies, from identification, sequencing, structural analysis, epitope mapping, determination of neutralization mechanism and identification of virus mutants and variants for further study. A future application would be to apply this technique to newly discovered spike domain escape mutant variations at amino acids 504 and 560 for HAstV-1, 564 and 565 for HAstV-2, and 464 and 597 for AstV8 [51]. Such studies would provide further knowledge on the mechanisms of AstV neutralization and development of targeted therapeutics against the virus.

## 8. Engineering and Practical Applications of AstV Structural Proteins and VLPs

As our understanding of AstV structural proteins expand, there is great potential for developing applied technologies using these proteins. One technology that can directly benefit from this information is the development of new astrovirus vaccines for either mammalian or avian hosts [51,52,53,54,55]. For example, one group fused the amino acids 423-630 from TAstV2 (i.e., approximately the first 2/3 of the TAstV2 spike domain) to the spike domains of a pig Hepatitis E and human norovirus to create a trivalent vaccine candidate [52]. The trivalent vaccine was found to form predominantly tetramers and less frequently dimers, an oligomerization behavior that may be complicated by the use of an incomplete TAstV2 spike domain. This system design takes advantage of the polymerization ability of enteric virus spike domains and their inherent stability to provide a robust immune response, along with a template for future AstV therapeutic designs. Other systems have begun to identify key antigenic regions of mammalian and avian AstV capsids that can be interdependently purified and used for producing an immune response, or new expression systems to produce those target proteins [53,54,55].

Recent developments in AstV reverse genetics systems allow for precision engineering and good recovery from expression cell lines [56,57]. Even with the availability of this system, there have not been any reported attempts to engineer the AstV proteins into a therapeutic or vector system. One candidate system would be an AstV VP70 capsid assembly with no trypsin cleavage sites. This would essentially generate a “locked” virus that would have a very limited ability to infect new host cells. From a basic science perspective, such a virus variant could allow for testing the properties of this stage of the virus, such as host factors that drive maturation of the viral capsid or if an entirely new form of mature capsid other than VP34/27/25 could be observed. In terms of applications, it may be possible to engineer this virus into a vaccine candidate that can present some of the conserved antigenic domains but have little to no risk of causing the activity or pathogenesis seen in the VP34/27/25 capsid. Similar systems have precedent in other viruses, such as norovirus capsid-derived nanoparticles [58].

The maturation process of the AstV capsid provides an interesting direction for protein and viral vector design. Rather than being mediated by viral proteases, the astrovirus capsid changes its structure and property throughout the virus life cycle in a process driven by the host environment. Other virus systems have been engineered to utilize host proteases in order to function, such as adeno-associated virus (AAV) derived genetic therapy vectors that only transduce cells when activated by tumor environments through metalloproteinases [59,60]. The AstV capsid is a prime candidate for adapting to an activatable viral system, as its capsid is already activated by the host environment. If the AstV capsid could be engineered to deliver a therapeutic cargo and have its maturation process factors specific to gastric diseases, such as changes in pH, proteases, or bile salts, then there is potential as a targeted therapy platform. 

## 9. Conclusions and Future Perspectives

Since the first discovery of human astroviruses in 1975, our understanding of the astrovirus capsid structures and functions has continued to expand through the years, especially in areas related to laying down the structural framework of the capsid protein and elucidating maturation-related structural changes as well as mechanisms of antibody neutralization. Exciting new insights have begun to emerge regarding astrovirus receptor binding, host entry, virus assembly and cellular exit, although a detailed mechanistic understanding is still lacking. Compared to other gastroenteric viral pathogens, HAstVs are certainly under-characterized at this time. 

Some of the most urgent questions involving the capsid that remain to be answered are, but not limited to: (1) the underlying mechanism that allows mature astrovirus to gain infectivity after extracellular protease processing of the viral capsid; (2) the identity of the cellular receptor for the virus and its interaction with the capsid; (3) the mechanism of astrovirus internalization and host membrane penetration mediated by the viral capsid and/or associated factors; (4) the interaction of VP90 with the viral genome and other viral/host factors that leads to the assembly of a precise T = 3 particle; (5) the structure and function of the acidic domain of the astrovirus CP; and (6) the mechanism of intracellular trafficking and non-lytic release of the astrovirus particle. Considering recent advances in cellular imaging, electron tomography, molecular labeling, mass spectrometry, genetics, and other techniques in structural and molecular biology, we are optimistic that accelerated progress will be made in various fronts. The discovery of neuropathogenic astroviruses such as MLB and VA will also likely catalyze more intensive research efforts in studying the fundamental virus infection mechanisms.
